# Complete Genome Sequence of *Streptococcus thermophilus* SMQ-301, a Model Strain for Phage-Host Interactions

**DOI:** 10.1128/genomeA.00480-15

**Published:** 2015-05-21

**Authors:** Simon J. Labrie, Denise M. Tremblay, Pier-Luc Plante, Jessica Wasserscheid, Ken Dewar, Jacques Corbeil, Sylvain Moineau

**Affiliations:** a Département de Biochimie, de Microbiologie, et de Bio-Informatique, Faculté des Sciences et de Génie, Groupe de Recherche en Écologie Buccale, Faculté de Médecine Dentaire, Félix d’Hérelle Reference Center for Bacterial Viruses, Université Laval, Québec, Canada; b Département de Médecine Moléculaire, Faculté de Médecine, Université Laval, Québec, Canada; c Department of Human Genetics, McGill University and Génome Québec Innovation Centre, Montréal, Canada

## Abstract

*Streptococcus thermophilus* is used by the dairy industry to manufacture yogurt and several cheeses. Using PacBio and Illumina platforms, we sequenced the genome of *S. thermophilus* SMQ-301, the host of several virulent phages. The genome is composed of 1,861,792 bp and contains 2,037 genes, 67 tRNAs, and 18 rRNAs.

## GENOME ANNOUNCEMENT

*Streptococcus thermophilus* is a low-GC Gram-positive bacterium widely used by the dairy industry to obtain high-quality fermented products, such as yogurt and cheeses (1). As such, it is a rare nonpathogenic streptococcal species (2). Another distinctive feature of *S. thermophilus* is its production of lactic acid from only a few sugars, including glucose, lactose, and sucrose (3). Selecting a suitable industrial *S. thermophilus* strain is a long process, encouraging extensive use and thorough characterization of the available strains (4).

*S. thermophilus* SMQ-301 is an industrial strain used to make cheese that is also sensitive to several virulent phages, including the reference phage DT1 (5). Phage infection of bacterial cultures is still the main risk factor for slowed milk fermentation, and the dairy industry relies on various strategies to control this phenomenon (4). *S. thermophilus* SMQ-301 has been used in several studies on phage biology (5–10), clustered regularly interspaced short palindromic repeat (CRISPR)-Cas systems (11–13), and sugar metabolism (3, 14, 15). It has a similar pulsed-field gel electrophoresis restriction profile (data not shown) to that of *S. thermophilus* LMD-9 (1,856,368 bp, 39.1% G+C content, 1,834 genes, two plasmids; GenBank accession no. NC_008532), which is also sensitive to DT1 (10).

The genome of *S. thermophilus* SMQ-301 was sequenced using MiSeq (Illumina) and PacBio (Pacific Biosciences) platforms. DNA extraction, library preparation, and assembly were performed as described previously (16). Briefly, DNA was purified using Genomic-tip 20/G columns, according to the manufacturer’s instructions. The genome was assembled into a single contig with the PacBio reads, according to the manufacturer’s instructions, and base calling accuracy was verified and corrected using Illumina reads aligned with BLAT (17) to the PacBio assembly. The Illumina and PacBio data were highly concordant, with the exception of 27 loci that were corrected with the Illumina reads. The genome of 1,861,792 bp has a G+C content of 39.1%. SMQ-301 contains no plasmids. The RAST annotation server (18) was used to annotate the genome, which encodes 2,037 proteins, 67 tRNAs, and 18 rRNAs.

We identified three CRISPR-Cas loci in the genome of *S. thermophilus* SMQ-301. According to the classification of CRISPR-Cas systems (19), two of the loci are type II-A systems (CRISPR 1 and CRISPR 3), while the CRISPR-Cas 2 locus is a type III-A system. Moreover, SMQ-301 has 16 spacers in CRISPR 1, 3 spacers in CRISPR 2, and 15 spacers in CRISPR 3. The last 10 spacers (3′) of CRISPR 1 are identical to those of LMD-9, which also has 16 spacers. Both strains have the same 3 spacers in CRISPR 2, and they share 5 spacers in CRISPR 3, while LMD-9 has only 8 spacers in this locus. The genome of SMQ-301 also encodes two type-I restriction-modification systems. Although prophage genes were detected by Phast (20), no complete prophage is present.

No known toxins were identified in the coding sequence of *S. thermophilus* SMQ-301 by the Web server VirulenceFinder (21) or by comparing the protein sequences with the VFDB (22) and DBETH (23) toxin databases using BLASTp (24). Finally, no known antibiotic resistance genes were found in the genome of SMQ-301 in comparison with the antibiotic resistance database (ARG-ANNOT) (25).

### Nucleotide sequence accession number. 

The complete annotated genome sequence of *S. thermophilus* SMQ-301 was deposited in GenBank under accession no. CP011217.
